# COVID-19: Focusing on the Link between Inflammation, Vitamin D, MAPK Pathway and Oxidative Stress Genetics

**DOI:** 10.3390/antiox12051133

**Published:** 2023-05-20

**Authors:** Jessica Cusato, Alessandra Manca, Alice Palermiti, Jacopo Mula, Martina Costanzo, Miriam Antonucci, Francesco Chiara, Elisa Delia De Vivo, Domenico Maiese, Micol Ferrara, Stefano Bonora, Giovanni Di Perri, Antonio D’Avolio, Andrea Calcagno

**Affiliations:** 1 Laboratory of Clinical Pharmacology and Pharmacogenetics, Department of Medical Sciences, University of Turin, Amedeo di Savoia Hospital, Corso Svizzera, 164, 10149 Turin, Italy; 2 ASL Città di Torino, Amedeo di Savoia Hospital, 10149 Turin, Italy; 3 Laboratory of Clinical Pharmacology S.Luigi A.O.U., Department of Clinical and Biological Sciences, University of Turin, Regione Gonzole, Orbassano, 10043 Turin, Italy; 4 Unit of Infectious Diseases, Department of Medical Sciences, University of Turin, Amedeo di Savoia Hospital, 10149 Turin, Italy

**Keywords:** SARS-CoV-2, MAPK, vitamin D

## Abstract

An uncontrolled inflammatory response during SARS-CoV-2 infection has been highlighted in several studies. This seems to be due to pro-inflammatory cytokines whose production could be regulated by vitamin D, ROS production or mitogen-activated protein kinase (MAPK). Several genetic studies are present in the literature concerning genetic influences on COVID-19 characteristics, but there are few data on oxidative stress, vitamin D, MAPK and inflammation-related factors, considering gender and age. Therefore, the aim of this study was to evaluate the role of single nucleotide polymorphisms in these pathways, clarifying their impact in affecting COVID-19-related clinical features. Genetic polymorphisms were evaluated through real-time PCR. We prospectively enrolled 160 individuals: 139 patients were positive for SARS-CoV-2 detection. We detected different genetic variants able to affect the symptoms and oxygenation. Furthermore, two sub-analyses were performed considering gender and age, showing a different impact of polymorphisms according to these characteristics. This is the first study highlighting a possible contribution of genetic variants of these pathways in affecting COVID-19 clinical features. This may be relevant in order to clarify the COVID-19 etiopathogenesis and to understand the possible genetic contribution for further SARS infections.

## 1. Introduction

Severe acute respiratory syndrome coronavirus-2 (SARS-CoV-2) was reported in Wuhan (China) for the first time on 31 December 2019 [1]. It caused the disease known as Coronavirus Disease 19 (COVID-19), which rapidly spread worldwide, resulting in a pandemic declared by the WHO (World Health Organization) on 11 March 2020 [2]. Since then, different waves have taken place and, currently, the disease seems to be toned down similar to a flu for the large majority of immune-competent patients [3]. Nevertheless, SARS-CoV-2 is still considered a severe infection for some populations, including elderly subjects, critically ill patients and patients with co-morbidities, such as diabetes, obesity, chronic obstructive pulmonary disease, etc. [3].

Therefore, research studies were carried out to understand the molecular mechanisms underlying COVID-19 in order to identify the specific clinical characteristics of critically ill patients and find therapeutic alternatives to minimize the severity of COVID-19 [4].

The WHO suggested the virus spreads mainly between people in close contact with each other, for example, at a conversational distance. The virus can spread from an infected person’s mouth or nose in small liquid particles when they cough, sneeze, speak, sing or breathe. Another person can then contract the virus when infectious particles that pass through the air are inhaled at short range [5].

This kind of disease involves the respiratory, gastrointestinal, cardiovascular and nervous systems, leading to different manifestations [6], ranging from non-severe, such as flu-like and gastrointestinal symptoms, to severe, such as renal failure, acute respiratory distress syndrome and death [7].

Acute respiratory distress syndrome is reported in 41.8% of patients with COVID-19. It is characterized by a “cytokine flare” with a systemic involvement, and it has been commonly reported to be associated with fatal outcomes [6,7]. In particular, SARS-CoV-2 causes the activation of different cells in the immune system, such as monocytes, macrophages and dendritic cells, resulting in the secretion of several cytokines and pro-inflammatory mediators, such as interleukin (IL)-28, IL-6, IL-10 and Tumour Necrosis Factor-alpha (TNF-α). Currently, the role of IL-28 in COVID-19 infection is not yet clarified. As a consequence of its variable expression rate and its important role in viral immunity, it was hypothesized that blood levels of IL-28 might be an indicator of the clinical outcome of the disease and it could be used for risk assessment in COVID-19 patients [8].

Concerning IL-6, it is secreted by macrophages as a pro-inflammatory cytokine. It is considered an important mediator of the acute phase reaction, and when it is deregulated, it could lead to the development of cytokine release syndrome (CRS). It is characterised by an extreme increase in the inflammatory response of multiple cytokines. This condition can be associated with systemic inflammation, multi-organ failure and, in some cases, death [9].

Several studies showed that high IL-10 expression levels predict negative outcomes in patients with COVID-19, and this appears to be a characteristic mark of hyper-inflammation during severe SARS-CoV-2 infection. IL-10 is basically categorized as an anti-inflammatory cytokine and rises dramatically early in the course of disease development [10,11].

Moreover, as reported in the study of Giamarellos-Bourboulis et al., the immune response of 54 COVID-19-affected patients with pneumonia led to an over-production of pro-inflammatory cytokines and, subsequently, B cell lymphopenia. In particular, it was reported that TNF-α and IL-6 production by circulating monocytes was sustained [12].

It is known that inflammation is modulated by different signalling pathways and mediators, including vitamin D and reactive oxygen species (ROS).

VD deficiency is a public health problem: it may be due to scarce sunlight exposure, skin pigmentation, black ethnicity, elderly age, low vitamin D intake, liver and kidney diseases, gastrointestinal malabsorption, diabetes mellitus, obesity and alcohol abuse [13].

Vitamin D is inactive and needs to be activated through two pathways known as a canonical, with sequential hydroxylation at C25 and C1α [14], and a non-canonical, activated by CYP11A1 [15,16,17].

In detail, the canonical pathway of vitamin D is activated by two steps thanks to the sun exposition: cutaneous 7-dehydrocholesterol is converted into pre-vitamin D3 after ultraviolet light exposure [13]. Cholecalciferol is hydroxylated to calcifediol by the cytochrome-450 (CYP) 27A1 and CYP2R1 in the liver, and calcitriol (1,25-dihydroxy vitamin D) is synthesized by CYP27B1 in the kidney. Afterwards, 1,25-VD is transported in blood through the vitamin-D-binding protein [18].

Regarding the non-canonical pathway, vitamin D activation starts with CYP11A1, a steroidogenesis enzyme, expressed in adrenals, placenta, gonads, in immune cells and skin. This is a complex process, involving a large number of CYPs and producing several hydroxyderivatives [19]. The CYP11A1-derived hydroxyderivatives are non-calcaemic or low calcaemic and can be used at high concentrations for therapeutic scopes: they can modulate gene expression by binding the receptor (VDR). Moreover, a bioinformatics approach reported that VDR could be used by other anti-inflammatory mediators, such as ghrelin, exerting an immunomodulatory function in the course of SARS-CoV-2 infection [20].

In addition, CYP11A1-derived hydroxyderivatives can bind other nuclear receptors, such as retinoic acid orphan receptors, aryl hydrocarbon receptor (AhR) and liver X receptors α and β, changing their expression and activities [19,21].

The 20(OH)D3 and 20,23(OH)2D3 hydroxymetabolites of vitamin D3 derived by CYP11A1 showed anti-inflammatory and anti-oxidative effects, as described for the classical active forms [19]. Several studies suggested that the vitamin D signalling pathway may provide some beneficial effects in lipoprotein-induced acute respiratory distress syndrome.

In fact, vitamin D is able to act through several mechanisms: for example, decreasing the storm of cytokines and chemokines; regulating the renin–angiotensin–aldosterone system; modulating neutrophil activity; maintaining the integrity of the pulmonary epithelial barrier; and stimulating epithelial repair [22].

In cancer, vitamin D has a role in inducing the expression of numerous enzymes involved in ROS detoxification, including superoxide dismutase 1 (SOD1) and 2 (SOD2) [23,24]. Furthermore, vitamin D induces the expression of thioredoxin reductase 1 (TXNRD1), which reduces both thioredoxin for its antioxidant function [23,25] and glucose-6-phosphate dehydrogenase (G6PD), which produces NADPH for glutathione (GSH) regeneration.

Recent studies highlighted how oxidative stress is an essential factor that increases the severity of COVID-19 in some patients, especially associated with pulmonary dysfunction and the cytokine storm or in viral sepsis derived from SARS-CoV-2 infection [4].

Furthermore, the vitamin D signalling pathway plays a central role in protecting cells from elevated mitochondrial respiration and associated damage and overproduction of ROS, which can lead to cellular and DNA damage [26].

As reported by Ricca et al. [26], the genetic silencing of VDR in three different cell types was carried out in order to test the VDR importance in cell physiology. The receptor ablation resulted in higher respiratory activity that increases intracellular ROS production.

VDR controls both the mitochondrial and the nuclear transcription of the proteins involved in respiratory chain and ATP synthesis, playing a key role in coordinating the nuclear and the mitochondrial transcription of the components of the respiratory process.

The respiratory chain is mainly involved in ROS generation. Their production is beneficial to some extent. In fact, they have a role in cell cycle progression. However, excessive ROS production can become toxic, leading to cell damage.

In this regard, a study by Ricca et al. suggested that an increase in ROS levels is remarkable when VDR is silenced, and it could exceed the antioxidant defences. Indeed, while the initial effect of silencing was to arrest the growth and the modulation of the cell cycle, the long-term effect of VDR loss was cell damage, measured as LDH release [26].

Furthermore, the mechanism underlying the link between oxidative stress and inflammation seems to be clarified in some studies [27]: higher ROS generation at the site of inflammation leads to endothelial dysfunction and tissue injury. Under these pathological conditions, oxidative stress promotes the opening of inter-endothelial junctions and the migration of inflammatory cells across the endothelial barrier. Inflammatory cells helping in the pathogens elimination and foreign particles are also involved in tissue injury [28].

A previous study highlighted that TNF-α, IL-1β and IFN-γ induce ROS production in retinal pigment epithelium cells. The TNF-α-induced ROS are produced in mitochondria, IL-1β-induced ROS are produced via NADPH oxidase and IFN-γ-induced ROS are generated by both mechanisms [29].

On the other hand, IL-6 seems to influence the physiological redox balance, for example, in the skeletal muscle [27]: free radical production is involved in the oxidative bad impact on cellular components, e.g., proteins and lipids. In addition, as reported by Amini et al., COVID-19 can promote oxidative stress through some important pathways, including TNF-α [30]. Focusing on vitamin D, it is known that it is able to reduce IL-1, IL-6 and TNF-α and, simultaneously, it increases anti-inflammatory IL-10, influencing T-helper 17 maturation towards T-cell populations [22,31]. In fact, vitamin D is very active against COVID-19, as reported in several articles [32,33]: this pro-hormone induces the conversion of monocytes to macrophages, modulating T- and B-cell activity. Mohan et al. highlighted the role of vitamin D in decreasing cytokine storm following innate immune response activation in COVID-19-infected patients [34].

Different studies report genetic factors predicting COVID-19-related features, but no data are available for genetics of oxidative stress, vitamin D, mitogen-activated protein kinase (MAPK) and inflammation-related factors. Therefore, the aim of this study was to analyse associations between different genetic variants and individual response to SARS-CoV-2 infection, in order to clarify their role as biomarkers in the prediction of COVID-19 severity.

## 2. Materials and Methods

### 2.1. Patient Enrolment

Subjects were enrolled at the Amedeo di Savoia Hospital in Turin, Italy, and samples were analysed in the lab of Clinical Pharmacology and Pharmacogenetics—Amedeo di Savoia Hospital (Turin, Italy) in 2020 (first and second COVID-19 pandemic waves). A molecular swab for SARS-CoV-2 detection and an ethylenediamine tetra-acetic acid (EDTA) tube for whole blood collection for genetic analysis were obtained. The study was performed in compliance with the Declaration of Helsinki and local review board regulations. The study was conducted after approval from the local Ethics Committee (“A.O.U. Città della Salute e della Scienza di Torino-A.O. Ordine Mauriziano di Torino–A.S.L. Città di Torino”, Protocol E-Covid, n. 00171/2020). The enrolled patients gave written informed consent, according to the local ethics committee standards.

### 2.2. SARS-CoV-2 Detection Methods

Viral RNA extraction was carried out using MagNA Pure compact instrument (Roche, Basel, Switzerland), then processed with a real-time polymerase chain reaction (RT-PCR) (CFX-96, Bio-Rad, Hercules, CA, USA) following the Liferiver Novel Coronavirus 2019-nCov Real-Time RT-PCR kit protocol for the N, E and ORF1ab genes (Liferiver Bio-Tech, San Diego, CA, USA). Positivity was considered when 3/3 or 2/3 genes were present before the 35 threshold cycle.

### 2.3. Genetic Polymorphism Analyses

DNA extraction was realized with the “QIAamp DNA mini kit” (Qiagen, Valencia, CA, USA). These kits contain columns allowing for DNA purification starting from 200 µL of blood or plasma. Allelic discrimination was assessed through the RT-PCR (CFX-96, BIORAD, Hercules, CA, USA).

The following allelic variants were analysed:

*VDR* ApaI C > A (rs7975232), *VDR* TaqI T > C (rs731236), *VDR* BsmI G > A (rs1544410), *VDR* FokI T > C (rs17535810), *VDR* Cdx2 A > G (rs11568820), *VDBP* GC1296 A > C (rs7041), *CYP27B1*-1260 G > T (rs10877012), *CYP24A1* 3999 T > C (rs2248359), *CYP24A1* 8620 A > G (rs2585428), *CYP27B1* + 2838 C > T (rs4646536), *CYP27A1* 345 A > G (rs4674345), *CYP2C8* 681 C > T (rs1059681) and *CYP24A1* 22776 C > T (rs927650), *IL-28* 860 T > C (rs12979860), *IL-28* 917 T > G (rs8099917), *IL-28* 275 G > A (rs12980275), *IL-10* 3575 T > A (rs1800890), *IL-6* 597 G > A (rs1800797), *HNF4* 975 C > G (rs1884613), *PXR* 63339 C > T (rs2472677), *CAR* 540 C > T (rs2307424), *ESR* Pvull C > T (rs2234693), *HIF-1* 438 G > A (rs12434438), *HAMP* 582 G > A (rs10421768), *AK4* C > T (rs1109374), *AKR1C3* G > C (rs1937840), *MAPK* 792 G > A (rs2283792), *RAF* 931 T > C (rs3729931), *ERK* 966 T > C (rs2266966), *FLT1* G > T (rs722503), *VEGF* 2578 A > C (rs69947), *ACE* 2350 A > G (rs4343), *TNF-α* 308 G > A (rs1800629), *NT5C2* 153 C > T (rs10883841), *MAU2* C > G (rs13964), *VCAM1* T > C (rs1041163), *MBL2* C > G (rs7096206), *NRP1* T > C (rs2065364), *CES-1* G > T (rs2244613).

### 2.4. Statistical Analysis

The Shapiro–Wilk test was used to test the normality. Non-normal variables were represented as median and interquartile range (IQR); categorical variables were expressed as numbers and percentages. Differences for continuous variables and genetic groups were assessed with Kruskal–Wallis and Mann–Whitney tests, considering *p*-value < 0.05 as statistically significant.

Spearman tests (SC, Spearman coefficient) were used to evaluate correlations. In conclusion, predictive power of the considered variables was evaluated through univariate and multivariate logistic regression analysis (*p*-value = *p*; odds ratio = OR; interval of confidence = IC 95%).

All the statistical tests were performed with IBM SPSS Statistics 28.0 for Windows (Chicago, IL, USA).

## 3. Results

Of the 160 participants, 139 (86.9%) tested positive for SARS-CoV-2 detection and 21 tested negative. Participant characteristics are summarised in Table 1.

Participants had a median age of 64 years (interquartile range 60.6–66.3 years), and 111 (72.1%) were male.

Considering the type of ventilation and the symptoms, no differences were highlighted for age and gender with the exception of Non-Invasive Ventilation, such as Continuous Positive Airway Pressure (CPAP) between < and >65 years (*p* ≤ 0.011).

Furthermore, genetic variants influencing the type of ventilation are reported in Table 2, also considering the ventilation type grouped in no oxygenation vs. cannula + ventimask vs. Non-Invasive Ventilation + reservoir + intubation. Differences according to age and gender with type of ventilation are available in Supplementary Tables S1 and S2.

The SNP role in affecting the symptoms (fever, cough and dyspnoea) was evaluated and is reported in Table 3.

In a sub-analysis focused on age (65 years), the following SNPs were found associated with symptoms: for patients aged <65 years, *IL-6* 597 GA/AA (*p* = 0.042) and *MAPK* 792 AA (*p* = 0.013) were related with fever, *CYP24A1* 22776 CT/TT (*p* = 0.012) and *VDR* BsmI GA/AA (*p* = 0.021) with cough and, finally, *CYP27B1* + 2838 TT (*p* = 0.025), *HNF4* 975 CG/GG (*p* = 0.044) and *PXR* 63396 CT/TT (*p* = 0.014) with dyspnoea.

For patients aged >65 years, *CYP24A1* 3999 TC/CC (*p* = 0.010) and *CYP24A1* 22776 CT/TT (*p* = 0.014) were associated with fever, *CYP27B1*-1260 GT/TT (*p* = 0.019) and *IL-28* 860 TC/CC (*p* = 0.025) and *IL-28* 275 GA/AA (*p* = 0.025) with cough and, finally, *ERK* 966 TC/CC (*p* = 0.010) with dyspnoea.

Instead, considering gender, for female patients, *GC* 1296 AC/CC (*p* = 0.011) and *CYP24A1* 3999 TC/CC (*p* = 0.011), *HNF4* 975 CG/GG (*p* = 0.046) and *VCAM1* CC (*p* = 0.011) were related with fever, *CYP24A1* 22776 TT (*p* = 0.012) and *CYP2C8* 681 CT/TT (*p* = 0.012 with cough and, finally, *VDR* ApaI AA (*p* = 0.033), *VDR* BsmI GA/AA (*p* = 0.033), *CYP24A1* 8620 AG/GG (*p* = 0.006), *IL-28* 917 TG/GG (*p* = 0.033), *HNF4* 975 GG (*p* = 0.047), *PXR* 63396 TT (*p* = 0.047) and *CAR* 540 TT (*p* = 0.033) with dyspnoea.

For male patients, *VDR* CdX 2 AG/GG (*p* = 0.020) and *MAPK* 792 AA (*p* ≤ 0.001) were related with fever, *CYP27B1* + 2838 TT (*p* = 0.033), *CYP24A1* 22776 CT/TT (*p* = 0.015), *HNF4* 975 CG/GG (*p* = 0.030), *MAPK* 792 AA (*p* ≤ 0.030) and *AKR1C3* GC/CC (*p* ≤ 0.015) with cough and, finally, *VDR* FokI TC/CC (*p* = 0.024), *CYP27B1*-1260 GT/TT (*p* = 0.015), *ESR* Pvull CT/TT (*p* = 0.028), *MBL2* GG (*p* = 0.006), *ERK* 966 TC/CC (*p* = 0.041) and *VEGF* 2578 AC/CC (*p* = 0.015) with dyspnoea.

Genetic, demographic and pharmacological factors able to predict the Non-Invasive Ventilation/CPAP/Intubation use were analysed in a logistic regression analysis table (Table 4): only the *AKR1C3* CC genotype remained in the final multivariate regression.

## 4. Discussion

To date, globally there have been 762,201,169 confirmed cases of COVID-19, including 6,893,190 deaths reported by the WHO [35].

These critical numbers suggest the importance of identifying factors and biomarkers involved in COVID-19 severity to reduce negative outcomes. Moreover, the importance of understanding the mechanisms of increasing oxidative stress and its role still need to be clarified.

Since its onset, identifying risk factors capable of predicting the disease outcomes has been crucial: older age, diabetes, chronic lung disease, cardiovascular complications, obesity and cancer have been associated with COVID-19 severity. Even taken together, these factors are not enough to explain the disease complexity. Therefore, the host genetic variants in different fields, including oxidative stress, were investigated in order to clarify their putative roles in COVID-19 susceptibility and severity [36,37].

In particular, polymorphisms of *ACE2* and transmembrane serine protease 2 (*TMPRSS2*), two genes encoding for important factors involved in host cell entry, have been explored in recent studies. A recently published paper by Hou et al. suggested possible associations of coding region variants of these two genes with COVID-19 severity, susceptibility and outcomes. In addition, *ACE2* polymorphisms were more likely to be associated with cardiovascular and pulmonary conditions in the African/African-American population by altering the angiotensin (AGT)-ACE2 pathway. *TMPRSS2* genetic variants are related to increased susceptibility to disease and for risk factors such as cancer [38].

Another group investigated the ACE2 protein-altering variations and highlighted how ACE2 variants exist in human populations. It may increase or decrease its affinity to SARS-CoV-2 S-protein and, thereby, render individuals more resistant or susceptible to the virus [39]. In the literature, different studies suggested the association of polymorphisms of genes encoding for the aforementioned inflammatory biomarkers and COVID-19 severity [40], such as IL-6, IL-10 and Erythrocyte Sedimentation Rate (ESR).

Concerning this issue, a previous study reported that polymorphisms in the *IL10* gene are linked to the severity of the infected patients. This study found that *IL10* gene polymorphisms rs1800871, rs1800872 and rs1800896 were linked to COVID-19 mortality in different SARS-CoV-2 variants in the Iranian population [41].

In this study, different vitamin D pathway-related genetic variants were found to be associated with some symptoms and the type of oxygenation, also focusing on age < or >65 years and gender. For example, SNPs in *CYP27B1* were associated with cough and dyspnoea. Vitamin D status was associated with clinical characteristics in patients with COVID-19. A recent review published by Bignardi et al. [42] reported a meta-analysis of 21 studies: they suggest vitamin D deficiency associated with mortality in the overall analysis, but not when it is adjusted for the vitamin D cut-off levels <10/12 ng/mL. Thus, reduced vitamin D exposure was not related to increased mortality when adjustments for confounders are considered. Molecular-docking-based virtual screening analyses report that vitamin D hydroxy metabolites are able to bind to the active sites of two SARS-CoV-2 transcription machinery enzymes (main protease (Mpro) and RNA-dependent RNA polymerase (RdRP)), important in viral replication and infection [43,44]. These authors tested the vitamin D3 and lumisterol analogue ability in inhibiting these enzymes, suggesting 25(OH)L3, 24(OH)L3 and 20(OH)7DHC are the most effective, causing Mpro 10–19% inhibition and RdRP by 50–60% [19,45].

On the other hand, as vitamin D is an important immune system modulator, different studies suggest vitamin D supplementation could be useful in order to prevent and/or treat COVID-19 [46]. Moreover, vitamin D is involved in oxidative stress protection: as suggested by Jeon et al. [47], the treatment of rats with vitamin D markedly reduces the levels of malondialdehyde, a lipid peroxidation product causing DNA damage [48]. Therefore, vitamin D daily supplementation in humans could have a role in decreasing oxidative DNA damage [49].

It is important to highlight that, since vitamin D has a role in attenuating the cytokine storm and inhibiting viral replication, some authors suggest it as an excellent candidate drug for COVID-19 and for their use as nutrients or supplements in the prevention of COVID-19 [19]. In this context, a study focused on the different routes of delivery of vitamin D precursors, which can have an important effect on the final circulating vitamin D derivatives [15,45]. Particularly, oral administration requires liver hydroxylation to 25(OH)D, without being recognized by CYP11A, which only acts on its precursor, vitamin D itself. This could lead to 30-times reduced levels of the 20(OH)D3 form in serum compared with the 25(OH)D3 form. On the other hand, adequate systemic (adrenal gland) or local (immune system) amounts of CYP11A1-derived vitamin D hydroxyderivatives would require parenteral delivery. This route could include intra-muscular, subcutaneous or intravenous injections, and CYP11A1-derived products would be mainly produced in the adrenal gland for systemic purposes. However, they can also be generated in peripheral tissues expressing CYP11A1, as skin. Finally, there is a link between vitamin D and MAPK, since the pro-hormone is able to inhibit the pathway [45].

Concerning the MAPK pathway, genetic variants in MAPK and ERK genes were associated with the type of ventilation and the symptoms in our study.

Finally, it is important to highlight that oxidative stress is able to induce the MAPK signalling pathway: ROS production can have both pro-survival and proapoptotic effects. ROS-activated PLC-gamma and Src phosphorylate Ras and Raf, which activate ERK; it both positively and negatively regulates ROS levels indirectly via induction of p22phox, leading to higher ROS production and activation of Nrf2, upregulating antioxidants [50]. The presence of a physiologic 25-hydroxy vitamin D concentration enhances the expression of the nuclear factor Nrf2 and enhancing Klotho, a phosphate-regulating hormone and also an antiaging protein [51]. Klotho also regulates cellular signalling systems, including the formation of antioxidants [52]. In animal studies, inefficient FGF23 and/or Klotho expression has been shown to cause premature aging and death [52].

Having suboptimal concentrations of serum 25-hydroxy vitamin D fails to subdue oxidative stress conditions, augment intracellular oxidative damage and the rate of apoptosis. The intracellular Nrf2 level is inversely correlated with the accumulation of mitochondrial ROS [53] and the consequent escalation of oxidative stress. Thus, Nrf2 plays a key role in protecting cells against oxidative stress. This mechanism is modulated by vitamin D [54].

*AKR1C3* GC/CC was found to be associated with cough in males, whereas the CC genotype with reservoir/CPAP is the only factor remaining in the final multivariate model of regression predicting the use of Non-Invasive Ventilation/CPAP/Intubation grade of ventilation.

This enzyme has been implicated in the metabolism of some antibiotics, including doxorubicin [55]. In addition, AKR1C3 is able to regulate the metabolism of a broad array of carbonyl-containing compounds, for example, it is involved in the activation of doxorubicin to its less active doxorubicinol metabolite. In addition, it seems to share the same properties as an oxidant enzyme in the alleviation of ROS accumulation in tumour cells [56,57].

In an article published by Wei et al., the authors suggested that an elevated expression of AKR1C3 could increase the resistance of cancer cells to ionizing radiation via the modulation of oxidative stress [57]: they explored the genetic alterations in non-radioresistant vs. resistant oesophageal cancer cells acquired due to long-term fractionated radiation, finding AKR1C3 more expressed in radioresistance-acquired cells. Furthermore, they suggested that AKR1C3 suppression restored the sensitivity of the acquired tumour cells and mice to ionizing radiation. Finally, they concluded that the cellular monitoring of the oxidative stress in the AKR1C3-elevated cells indicated that ionizing radiation-induced ROS accumulation and the concomitant DNA damage were significantly reduced, and every protective consequence disappeared upon AKR1C3 knockdown. These findings could highlight the potential involvement of AKR1C3 in reducing cellular ROS levels.

*AKR1C3* GG was found to be associated with haematological toxicity in breast cancer patients: they developed significantly reduced absolute leukocyte and neutrophil counts on day 15 compared to C-allele patients with a longer progression-free and overall survival. The authors suggested that the GG genotype of this non-coding intronic variant could influence AKR1C3 activity; thus, this could have an impact on its antioxidant action, probably leading to Non-Invasive Ventilation/CPAP/Intubation use, but further studies are needed in order to clarify these aspects.

The limitations of this study include a relatively small cohort of COVID-19-affected patients and some lacking information, including the clinical outcome (death or not) after the subacute/acute intensive care unit admission. In addition, AKR1C3 activity or quantification must be evaluated in further studies in order to clarify its role in COVID-19.

## 5. Conclusions

In conclusion, this is the first study showing a possible impact of some oxidative stress, vitamin D, MAPK and inflammation-related factors on some COVID-19-associated features. These data could be useful in order to clarify the ethiopatogenesis of this disease and the possible genetic contribution for this one and further SARS-CoV-2 infection.

## Figures and Tables

**Table 1:** Participant characteristics.

Characteristics of Enrolled Patients	
Number of Patients (n)	160
Positive COVID-19, n (%)	139 (86.9%)
Age (years), median (interquartile range)	64 [60.6–66.3]
Age > 65 years (%)	43.9%
Male sex (%)	111 (72.1%)
ICU, n (%) (available for 121 patients)	15 (12.4%)
Sub-acute, n (%) (available for 121 patients)	5 (4.1%)
Fever, n (%)	73 (45.6%)
Cough, n (%)	49 (30.6%)
Dyspnoea, n (%)	43 (26.9%)
No oxygenation, n (%)	5 (7.4%)
Cannula, n (%)	20 (29.4%)
Ventimask, n (%)	11 (16.2%)
Reservoir, n (%)	2 (2.9%)
CPAP, n (%)	23 (33.8%)
Intubation, n (%)	7 (10.3%)

ICU: Intensive Care Unit; CPAP: Continuous Positive Airway Pressure.

**Table 2:** Statistically significant influence (*p*-values) of genetic variants on the type of ventilation and considering ventilation-type groups (no oxygenation vs. cannula + ventimask vs. non-invasive ventilation + reservoir + intubation).

	No Oxygenation	Cannula	Reservoir	CPAP	Intubation	Ventimask	No Oxygenation vs. Cannula/Ventimask vs. NIV/INT/Reservoir
*IL-6* 597 GA/AA		0.019					
*IL-28* 275 GA/AA	0.010						0.023
*IL-28* 275 AA		0.008					
*IL-28* 860 CC	0.020						
*IL-28* 917 GG			0.007				
*IL-10* 3575 AA				0.040			
*CYP24A1* 399 TC/CC							0.012
*CYP27A1* 345 AG/GG					0.029	0.042	
*HAMP* 582 GA/AA	<0.001						<0.001
*HIF-1* 438 AA			0.007				
*AKR1C3* CC			0.021	0.011			
*MAPK* 792 GA/AA				0.019			0.044
*VCAM1* TC/CC					0.012		
*MBL2* CG/GG					0.003		
*MBL2* GG							0.031
*PXR* 63396 TT					0.026		
*CAR* 540 CT/TT					0.019		

NIV: non-invasive intubation; CPAP: Continuous Positive Airway Pressure; INT: Intubation.

**Table 3:** Participant’s principal symptoms according to genetic influence (*p*-values).

SNPs	Symptoms		
	Fever	Cough	Dyspnoea
*VDR* BsmI GA/AA			0.032
*VDR* FokI TC/CC			0.027
*CYP27B1* + 2838 TT		0.010	0.013
*CYP27B1*-1260 GT/TT		0.039	0.044
*MAPK* 792 AA	<0.001		
*ERK* 966 TC/CC			0.021
*ESR* Pvull CT/TT			0.028
*MBL2* GG			0.021
*VEGF* 2578 AC/CC			0.042

SNPs: single nucleotide polymorphisms.

**Table 4:** Univariate logistic regression analysis for predictors of non-invasive intubation (NIV)/Continuous Positive Airway Pressure (CPAP)/Intubation (INT) ventilation.

	Univariate Regression	
Predictive Factors	*p*-Value	OR (95% IC)
Heparin	0.795	0.857 (0.267;2.747)
Chloroquine/Hydroxychloroquine	0.091	2.694 (0.855;8.491)
Lopinavir/D	0.394	1.704 (0.500;5.800)
Steroids	0.155	0.450 (0.150;1.352)
Remdesivir	0.117	0.262 (0.049;1.398)
Tocilizumab	**0.011**	6.356 (1.527;26.453)
Azithromycin	0.213	2.600 (0.578;11.693)
Ceftriaxone	0.893	0.913 (0.242;3.437)
Others ATB	0.468	0.625 (0.175;2.227)
Gender	0.268	0.545 (0.187;1.594)
Age > 65	0.102	2.267 (0.850;6.045)
BMI > 25	NC	
*VDR* ApaI AA	0.422	0.624 (0.197;1.974)
*VDR* TaqI CC	0.907	0.926 (0.253;3.383)
*VDR* BsmI AA	0.848	0.857 (0.174;4.164)
*VDR* FokI CC	0.751	0.855 (0.326;2.243)
*VDR* Cdx2 GG	0.751	1.169 (0.446;3.067)
*GC* 1296 CC	0.401	1.591 (0.538;4.706)
*CYP27B1*-1260 GT/TT	0.537	0.733 (0.274;1.965)
*CYP24A1* 3999 CC	**0.037**	0.222 (0.054;0.914)
*CYP24A1* 8620 GG	0.581	0.720 (0.224;2.314)
*CYP27A1* 345 GG	0.333	0.583 (0.196;1.736)
*CYP27B1* + 2838 TT	0.375	1.571 (0.579;4.267)
*CYP24A1* 22776 TT	0.300	0.500 (0.135;1.852)
*CYP2C8* 681 CT/TT	0.399	1.667 (0.509;5.461)
*IL-28* 860 CC	0.332	1.607 (0.616;4.194)
*IL-28* 917 GG	0.903	1.133 (0.150;8.548)
*IL-28* 275 GA/AA	0.822	0.867 (0.249;3.017)
*IL-6* 597 GA/AA	0.346	1.587 (0.608;4.143)
*IL-10* 3575 AA	0.084	0.409 (0.148;1.128)
*MAPK792* GA/AA	0.024	0.200 (0.049;0.810)
*RAF* 931 CC	0.585	1.481 (0.361;6.064)
*ERK* 966 TC/CC	0.754	0.846 (0.298;2.404)
*AKR1C3* CC	0.038	0.291 (0.091;0.934)
*AK4* CT/TT	0.567	0.733 (0.254;2.121)
*HNF4* 975 GG	0.326	2.429 (0.414;14.251)
*PXR* 63396 CT/TT	0.942	1.046 (0.311;3.515)
*CAR* 540 TT	0.859	1.143 (0.261;4.999)
*ESR* Pvull CT/TT	0.788	0.857 (0.279;2.631)
*CES1* GT/TT	0.120	2.200 (0.815;5.937)
*NT5* 153 TC/CC	0.441	0.592 (0.156;2.246)
*FLT1* TT	0.313	0.413 (0.064;2.297)
*MBL2* GG	0.010	0.259 (0.093;0.725)
*HIF-1* 438 GA/AA	0.318	0.612 (0.234;1.602)
*VEGF* 2578 AC/CC	0.582	0.724 (0.229;2.286)
*VCAM1* TC/CC	0.048	3.336 (1.011;13.083)
*MAU2* GG	0.583	1.440 (0.392;5.284)
*NRP1* TC/CC	0.460	1.442 (0.546;3.807)
*ACE* 2350 GG	0.705	0.818 (0.289;2.315)
*TNF* 308 GA/AA	0.632	0.761 (0.248;2.331)
*HAMP* 582 AA	0.523	0.722 (0.266;1.960)

NIV: non invasive intubation; INT: Intubation; ATB: Antibiotics; BMI: body mass index. Bold is used for statistically significant data (*p* < 0.05).

## Data Availability

Data are contained within the article.

## Supplementary Materials

The following supporting information can be downloaded at: https://www.mdpi.com/article/10.3390/antiox12051133/s1, Table S1: Influence of genetic variants on ventilation type according to age; Table S2: Participant type of ventilation according to genetics and gender.
