# Supplementary material for: COVID-19: Focusing on the Link between Inflammation, Vitamin D, MAPK Pathway and Oxidative Stress Genetics
Source: Antioxidants (Basel). 2023 May 20;12(5):1133. doi: 10.3390/antiox12051133 (PMC10215473; doi:10.3390/antiox12051133)
Supplement: Supplementary file 1 [file antioxidants-12-01133-s001.zip › antioxidants-2348914-supplementary.pdf]

Table S1. Influence of genetic variants on ventilation type according to age.

	<65 years (0)							>65 years (1)						
	No oxygenation	Cannula	Reservoir	CPAP	Intubation	Ventimask	No oxygenation vs. Cannula/Ventimask vs. NIV/Intubation/Reservoir	No oxygenation	Cannula	Reservoir	CPAP	Intubation	Ventimask	No oxygenation vs. Cannula/Ventimask vs. NIV/Intubation/Reservoir
**IL-6 597 GA/AA**		0.048												
**IL-6 597 AA**						0.046								
**IL-28 275 GA/AA**								0.046					0.038	
**IL-28 275 AA**	0.030	0.048												
**HAMP 582 GA/AA**	0.006						0.010							
**HIF-1 438 AA**										<				
**AKR1 CC**											0.017			0.039
**ACE 2350 AG/GG**			0.018					0.029						0.034
**IL-10 3575 AA**			0.009										0.012	
**MBL2 CG/GG**					0.008									
**NT5C2 153 TC/CC**								0.011						0.039
**NT5C2 CC**				0.042										
**NRP1 TC/CC**														
**IL-28 860 CC**	0.023													
**IL-28 917 GG**	0.003						0.011			0.003				
**PXR 63396 TT**					0.036									
**CAR 540 CT/TT**					0.035									
**CYP24A1 3999 TC/CC**	0.047		0.019								0.004			
**CYP24A1 3999 CC**								0.046						
**CYP24A1 8620 GG**								0.026						
**CYP24A1 8620 AG/GG**	0.048										0.009			

**CYP27A1 345 GG**									0.009					0.039
**CYP27A1 345 AG/GG**					0.022									
**CYP 2C8 681 CT/TT**										0.011				
**CYP27B1+2838 TT**				0.032										
**VEGF 2578 CC**									0.009					
**VEGF 2578 AC/CC**													0.006	
**VDR ApaI AA**								0.026		0.026				
**VDR TaqI CC**										0.003				
**VCAM1 TC/CC**					0.036					0.026				
**MAU 2 GG**			0.018											
**RAF 931 CC**			0.018					0.003						0.011
**ERK 966 CC**								0.026						

Table S2. Participant type of ventilation according to genetics and gender.

	Female (0)							Male (1)						
	No oxygenation	Cannula	Reservoir	CPAP	Intubation	Ventimask	No oxygenation vs. Cannula/Ventimask vs. NIV/Intubation/Reservoir	No oxygenation	Cannula	Reservoir	CPAP	Intubation	Ventimask	No oxygenation vs. Cannula/Ventimask vs. NIV/Intubation/Reservoir
**AKR1 ET MT**									0.036					
**VEGF 2578 CC**		0.033												
**RAF 931 TC/CC**						0.012	0.046							
**IL-10 3575 AA**				0.013		0.012	0.046							
**HAMP 582 GA/AA**								<0.001						<0.001
**AKR1 CC**											0.012		0.016	0.010
**HIF-1 438 AA**										<0.001				
**IL-10 3575 TA/AA**			0.047											
**VCAM1 TC/CC**					0.047					0.046				
**MAPK 792 GA/AA**											0.047			0.046
**MBL2 CG/GG**												0.007		
**HIF-1 438 GA/AA**												0.043		
**IL-28 860 CC**								0.012						0.040
**IL-28 275 GA/AA**								0.005						
**GC 1296 AC/CC**		0.028									0.021			0.038
**IL-28 275 AA**									0.047					
**IL-6 597 GA/AA**									0.024					
**CYP27A1 345 AG/GG**													0.043	
**CAR 540 CT/TT**						0.028						0.018		
**VDR TaqI CC**										0.046				
**CYP24A1 3999 TC/CC**			0.003											0.026
**IL-28 917 GG**										<0.001				
**PXR 63396 CT/TT**			0.018											
**HNF4 975 GG**				0.047										
**VDR ApaI AA**					0.018									
**CYP24A1 8620 GG**					0.003									
**CYP27A1 345 AG/GG**					0.018									
**CYP24A1 22776 TT**					0.018									
**PXR 63396 TT**												0.041		
**IL-28 IL-28 275 GA/AA**														0.018
